# Predicting secondary school dropout among South African adolescents: A survival analysis approach

**DOI:** 10.15700/saje.v37n2a1353

**Published:** 2017-05

**Authors:** Elizabeth H. Weybright, Linda L. Caldwell, Hui (Jimmy) Xie, Lisa Wegner, Edward A. Smith

**Affiliations:** Human Development Department, College of Agricultural, Human, and Natural Resource Sciences, Washington State University, Pullman, Washington, USA; Department of Recreation, Park, and Tourism Management, College of Health and Human Development, The Pennsylvania State University, University Park, Pennsylvania, USA; Faculty of Community and Health Sciences, University of the Western Cape, Bellville, South Africa; Department of Recreation and Tourism Management, College of Health and Human Development, California State University, Northridge, California, USA; Department of Occupational Therapy, Faculty of Community and Health Sciences, University of the Western Cape, Bellville, South Africa; Edna Bennett Pierce Prevention Research Center, College of Health and Human Development, The Pennsylvania State University, University Park, Pennsylvania, USA; Faculty of Community and Health Sciences, University of the Western Cape, Bellville, South Africa

**Keywords:** adolescence, leisure motivation, school dropout, substance use

## Abstract

Education is one of the strongest predictors of health worldwide. In South Africa, school dropout is a crisis where by Grade 12, only 52% of the age appropriate population remain enrolled. Survival analysis was used to identify the risk of dropping out of secondary school for male and female adolescents and examine the influence of substance use and leisure experience predictors while controlling for demographic and known predictors using secondary, longitudinal data. Results indicated being male, not living with one’s mother, smoking cigarettes in the past month, and lower levels of leisure-related intrinsic motivation significantly predicted dropout. Results support comprehensive prevention programmes that target risk behaviour and leisure.

## Introduction

Education is one of the strongest predictors of health worldwide (Freudenberg & Ruglis, 2007) with well-documented positive outcomes. Youth education is a global priority and given this, school dropout remains an urgent concern. Although international rates of dropout differ, one consistent finding is that dropping out of school results in poorer psychological, physical, social, and economic health (Lamb & Markussen, 2011).

In South Africa, dropout has reached a national crisis. Approximately 60% of first graders will ultimately drop out rather than complete 12th Grade. Likewise, by Grade 12, only 52% of the age appropriate population remain enrolled (Department of Basic Education, Republic of South Africa, 2015). In order to prevent learners from leaving school, we need to better understand why they are leaving school and what approaches may be effective in retaining them.

Cross-sectional studies consistently find dropout youth more likely to engage in risk behaviours including use of tobacco, alcohol, marijuana, and other drugs (Townsend, Flisher & King, 2007). However, results from longitudinal studies are not as clear, suggesting that there are other factors associated with school dropout. A unique contribution of this study is its inclusion of leisure-related variables, which to our knowledge is overlooked in studies examining school leaving. Other research has indicated that healthy leisure can be a protective factor and mitigate the use of substances and engagement in other risk behaviour. At the same time, however, leisure is also a context for participating in risky behaviour. The corpus of this research indicates that leisure motivation and leisure boredom are important aspects of understanding adolescent behaviour from a risk and protective factors perspective. Thus, we queried whether leisure motivation, leisure boredom and substance use are associated with dropout. To address this query, we used secondary, longitudinal data to look at how substance use and leisure experience contributed to school dropout while controlling for demographic factors and educational attainment that have been previously associated with school dropout.

### School Dropout

A report from South Africa’s Department of Basic Education (2011b) found an increase in school leaving across grades such that 6.5% of learners dropped out in Grade Nine but 11.5% and 11.8% dropped out in Grades 10 and 11, respectively. It is estimated that out of each 100 learners that begin school in Grade One, half will dropout, 40 will successfully complete the NSC exam, and only 12 will be eligible to pursue higher education (Lamb & Markussen, 2011).

Historically, one way low performance has been addressed is by holding learners back to repeat a grade. By the time learners reach Grades 10–12, 52% have repeated a grade and 9% of 12th Graders repeat a grade three times or more. This approach is not effective at graduating learners as “academic gains from retention tend to disappear or see a washout effect several years later” (Hickman, Bartholomew, Mathwig & Heinrich, 2008:4). In 2012, the Department of Basic Education, Republic of South Africa approved revised regulations stipulating learners “may only be retained once … [between Grades 10–12] in order to prevent the learner being retained in this phase for longer than four years” (2012:16). These new regulations mean learners that would have previously been retained more than once are instead automatically moving on to the next grade (except for Grade 12). New regulations may not be effective either given some have termed grade repetition the “most powerful predictor of dropout status” regardless of the number of times a learner has been held back (Jimerson, Anderson & Whipple, 2002:443).

### Conceptualising Dropout

The United Nations Educational, Scientific and Cultural Organization (UNESCO) Institute for Statistics defines dropout as the “proportion of pupils from a cohort enrolled in a given grade at a given school year who are no longer enrolled in the following school year” (2009:44). In South Africa, the Department of Education defines dropout as leaving school before completing a given grade in a given school year (Wegner, Flisher, Chikobvu, Lombard & King, 2008).

Although one-time events (e.g. family move) may contribute, in reality, school dropout is much more complex and has been considered a gradual process, suggesting dropping out may have a temporal pattern associated with it. Using in-depth interviews, Ananga (2011) classified dropout into two main categories of temporary and permanent dropout and within those categories, found evidence of different temporal patterns of school leaving. For example, sporadic dropout was characterised by intermittent school leaving for a few months and then returning to school. Learners classified as event dropouts had family, school, or life events (e.g. pregnancy) that caused them to dropout for long periods of time. Some of these learners would eventually go back to school but some would not. Youth who were classified as permanent dropout had no intention of going back to school. Some felt lost after dropping out but held open the possibility of returning to school if something in their context changed. Others left school because they could see no value in it or struggled and left to pursue a type of vocational training. Within the current study, we followed Ananga’s (2011) typology of permanent dropout in an attempt to more accurately capture those that have fully disengaged from the educational system.

### Determinants and Correlates of Dropout

Neither school dropout nor academic success is determined by the learner alone. From an ecological perspective, there are contributing multi-level and cross-level influences. These influences are found at the individual (e.g., gender, race, substance use (Townsend et al., 2007); previously failing a grade (Battin-Pearson, Newcomb, Abbott, Hill, Catalano & Hawkins, 2000), family (e.g., family composition; Ananga, 2011), and social level (e.g., poverty; Strassburg, Meny-Gilbert & Russell, 2010). Compiled results of a national household survey (a representative sample of 4,498 households) and focus groups with learners, parents, and educators identified four main reasons why learners left school. These included household poverty and cost of education (i.e. access costs), teenage pregnancy, a lack of interest in schooling, and previously failing a grade or being behind in school work (Strassburg et al., 2010). Given this, the current study controls for demographic factors including gender and race, academic achievement, family composition, and socio-economic status, which then allows us to focus on the two main factors of interest to this study, substance use and leisure experience.

### Substance Use

Research on the connection between dropout and substance use finds mixed results. Some research suggests dropouts initiate use at an earlier age and demonstrate greater intensity of use (Gasper, 2011). This association has been found in cross-sectional studies of SA adolescents, where dropouts exhibited greater use of tobacco, alcohol, and illegal substances (e.g., Flisher, Townsend, Chikobvu, Lombard & King, 2010). However, using longitudinal data from 8th Graders, Flisher and colleagues found only tobacco to be directly associated with dropout and not alcohol or marijuana, suggesting that the snapshots of use obtained by cross-sectional data may not accurately capture substance use behaviours.

Townsend et al. (2007) conducted a systematic literature review addressing the relationship between dropout and substance use including tobacco and alcohol. Supported by both cross-sectional and longitudinal studies, use of tobacco was consistently associated with dropout even after controlling for known covariates (e.g. gender, race, age) (Townsend et al., 2007). Youth at-risk for dropout tended to be heavier cigarette smokers and began smoking at an earlier age than their low-risk peers. However, support for the association between other substance use and dropout is less clear and studies have found mixed results.

At the conclusion of their review, Townsend et al. (2007) called for more research to better identify not only inter-dependent risk factors of dropout, but also protective factors, a topic which has been under-researched. They acknowledged the need for research in developing countries, suggesting results from developed countries may not directly apply to developing countries that “appear to have the least favorable school outcomes”, and yet also lack sufficient research to fully understand the dropout experience (2007:315).

### Leisure Experience

Leisure is a crucial developmental context for adolescents (Larson, 2000; Verma & Larson, 2003) and as such may serve as a protective factor. Leisure is one of the under-researched topics in school dropout that has relevance to the SA context. Engagement in healthy leisure may protect adolescents from negative outcomes such as deviant behaviour (Mahoney, 2000; Weybright, Caldwell, Ram, Smith & Jacobs, 2014) and increase adolescents’ self-esteem, academic performance, peer-group affiliation, and school engagement (Eccles & Barber, 1999; Eccles, Barber, Stone & Hunt, 2003; Mahoney, 2014), all of which in turn may reduce the likelihood of dropping out.

Using Self-determination Theory (Ryan & Deci, 2000) and Optimal Arousal Theory (Iso-Ahola, 1980) as the foundation for our work allows us to conceptualise why leisure experience might be associated with dropout. One of the reasons leisure might be healthy is because experientially, youth feel positive when engaged in meaningful and personally rewarding activities. In these situations, youth typically are not bored and feel more intrinsically motivated. Self-determination theory posits intrinsic motivation, an inherent tendency to engage in activities due to interest and personal satisfaction is associated with enjoyment, engagement, and healthy youth development (Ryan & Deci, 2000), while amotivation (non-intentionally motivated behaviour) and extrinsic motivation (behaviour motivated to meet external demands) often are associated with negative leisure experience and outcomes (Patterson, Pegg & Dobson-Patterson, 2000).

When youth do not have positive experiences in leisure such as when they are bored, negative outcomes are likely to occur. It is of particular concern when young people experience boredom in leisure and do not have the skills or motivation to change what they are doing into something more interesting. Grounded in Optimal Arousal Theory (Iso-Ahola, 1980), boredom in leisure has been linked to risk behaviour such as substance use and sexual risk behaviours in qualitative (Wegner, 2011), cross-sectional (Wegner & Flisher, 2009), and longitudinal studies (Miller, Caldwell, Weybright, Smith, Vergnani & Wegner, 2014; Weybright, Caldwell, Ram, Smith & Wegner, 2015). An adolescent’s ability to restructure boredom into something more interesting is an important developmental skill (Caldwell, Baldwin, Walls & Smith, 2004). Some research has shown that youth who do have the skills to restructure their experience into something more interesting are more likely to engage in healthy behaviours rather than in risk behaviour (Weybright et al., 2014). When looking at boredom within the school context, general levels of boredom have also been associated with academic disengagement (Strassburg et al., 2010).

### The Current Study

The current study sought to better understand the occurrence of dropout. Making use of secondary data consisting of eight waves of data between Grade Eight and Grade 11, we used survival analysis to identify the risk of dropping out for both male and female adolescents and examined the influence of substance use and leisure experience on high school learner dropout while controlling for demographic and known predictors. Survival analysis is a commonly used statistical method for not only describing the timing of an event, but also modelling the risk of an event’s occurrence and the influence of predictors over time (Singer & Willett, 2003). We hypothesised that: 1) males would have a higher hazard function (i.e. instantaneous risk that dropout will occur at a given time point) than females; 2) substance use will be significantly associated with increased dropout risk after controlling for demographic and known risk factors; and 3) leisure experience will be significantly associated with dropout risk (increased risk for boredom, amotivation, and extrinsic; decreased risk for intrinsic motivation) after controlling for substance use and demographic factors.

## Methods

### Study Setting, Participants, and Procedures

The current study used data drawn from a school-based sample of learners in Mitchell’s Plain, a low-income residential area approximately 15 miles outside of Cape Town, South Africa, who participated in an effectiveness trial of HealthWise South Africa, a leisure-based life skills curriculum intervention addressing adolescent health risk behaviour in a school setting (see Caldwell, Smith, Wegner, Vergnani, Mpofu, Flisher & Mathews, 2004). The Mitchell’s Plain geographical area was targeted due to its homogeneous context and schools were selected based on their degree of school organisation and cooperation, which facilitated conduct of the study. This homogeneity controlled for factors such as socio-economic status and contextual factors in the environment making it more feasible to identify outcomes.

Four schools were randomly assigned to receive the curriculum, and five schools were chosen as matched no-treatment control schools. The study and its passive parental consent and adolescent assent procedures were approved by the Institutional Review Board at the Pennsylvania State University, the Research Ethics Committee at Stellenbosch University, and by the Metro South Education District.

Learners were followed from the beginning of Grade Eight to the end of Grade 11 with data collected on eight occasions spaced six-months apart between March 2004 and October 2007. Learners completed surveys during school hours for approximately 30 minutes using a handheld digital device. The survey was available in both English and Afrikaans and administered in the learner’s home language. Research staff was available at each survey administration to answer questions or assist with difficulties.

For the present analysis, control group learners who demonstrated distinct patterns of school attendance were included. These 601 learners (50.9% female) ranged in age from 12–17 years old at baseline (Wave 1, *M* = 13.9, *SD* = 0.78), mostly reported their race as Coloured (91%; mixed ancestry), with few identifying as Black (6%), and Other (3%). Socio-economic indicators were consistent within the sample with 95% having running water, 97% electricity, and 82% residing in a brick house or flat. This homogeneity in socio-economic indicators is expected given that the sample came from the same geographic region.

### Measures

Measures in the current study included school dropout, substance use, subjective leisure experiences, control variables, known predictors of academic achievement, and demographic variables.

#### School dropout

School dropout was identified based on the pattern of participation in the school-based survey. As we will discuss further in the limitations section, using this method as a proxy of dropout has some complications, but given that we obtained comparable data to other studies that focused on SA dropout, we felt comfortable using this strategy. Two patterns were targeted for analyses including a *Complete* group and a *Dropout* group. The Complete group represented learners who were present and participated in all eight bi-annual measurement occasions from Grade Eight to 11 (i.e. XXXXXXXX where X = present). The Dropout group included those present for at least two initial measurement occasions in Grade Eight and not present for at least the two final measurement occasions in Grade 11 (i.e. XX----OO; where X = present and O = absent). Intermittent participators were excluded; for example, if a learner was present for the beginning and end of Grade Eight, missing for the beginning of Grade 9, and returned at the end of Grade Nine, they were excluded from analyses. Again, this decision was informed by Ananga’s (2011) findings regarding the temporal pattern of dropout. For this study, we were interested in those who demonstrated a strong pattern of permanent dropout status.

#### Substance use

Substance use was measured as past month use of alcohol and tobacco at each survey administration. Learners were asked “during the past four weeks, did you use alcohol/smoke cigarettes?” Responses were dichotomised (0 = no past month use). Past month use of alcohol and tobacco were entered as time-varying predictors.

#### Leisure experience

Subjective perceptions of leisure (i.e. boredom, amotivation, intrinsic, and extrinsic) were measured at each survey administration (see Table 1). All items had five response options ranging from “strongly disagree” to “strongly agree.” All four scales were included as time-varying predictors.

### Leisure boredom

Leisure boredom was measured using three items (e.g. “for me, free time drags on and on”) from the boredom subscale of the Leisure Experience Battery for Adolescents (LEBA; Caldwell, Smith & Weissinger, 1992; Iso-Ahola & Weissinger, 1990). After demonstrating reliability (Cronbach’s α = 0.68; equivalent in reduced leisure boredom subscale from LEBA; Caldwell et al., 1992), responses were averaged to obtain a summary leisure boredom index with higher scores indicating higher levels of boredom.

### Leisure motivation

Based on Self-Determination Theory (Ryan & Deci, 2000), motivation items used subscales of the Free Time Motivation Scale for Adolescents (FTMS-A; Baldwin & Caldwell, 2003) related to amotivation (three items; “I don’t know why I do my free time activities and I don’t really care”), external motivation (three items: “I do what I do in my free time because that is the rule in my house”), introjected motivation (two items; “I do what I do in my free time because I want to impress my friends”), identified motivation (four items; “I do what I do in my free time because it is important to me”), and internal motivation (three items; “I do what I do in my free time because I like what I do).

Recent empirical studies found a model with more concise structure may better reflect adolescents’ conceptual understanding and perception of leisure motivations (Sharp, Caldwell, Graham & Ridenour, 2006; Xie, Caldwell, Graham, Weybright, Wegner & Smith, 2016; Younker, Caldwell, Coffman & Smith, 2008). Given this, items were combined on external and introjected motivation (5 items) to represent adolescents’ extrinsic leisure motivation and combined items on identified and intrinsic motivation (seven items) to measure adolescents’ intrinsic motivation. Scales for amotivation, intrinsic, and extrinsic motivation demonstrated adequate reliability (Cronbach’s α = 0.81, 0.86, and 0.83 respectively) and responses were averaged, where higher scores indicated higher levels of each construct.

#### Control variables

Gender, living with a parent, previously failing a grade, and number of days absent from school were included as control variables as measured in Wave 1. Descriptives are included within Table 1. Gender was measured as a dichotomous variable (0 = male). Living with a parent was measured with two questions: “Does your mother/father live with you?” where mother and father were asked as separate questions. Responses were dichotomous (0 = no). Previously failing a grade was measured by “Have you ever failed a grade?” If the learner reported failing a grade at any measurement occasion, this was coded as yes (1), while never failing a grade was coded as no (0). Number of days absent from school was measured with “How many days were you absent from school during the last term?” where learners could enter a number between 0 and 100. The maximum number of absences reported was included in analyses.

### Analytic Strategy

Survival and hazard functions were estimated using the Kaplan-Meier (KM) estimator with SAS PROC LIFETEST. The survival function, or rate, is the probability that a learner survives longer than time t. This allows for identification of probabilities of survival at each time t, or Wave. The hazard function, or rate, gives the instantaneous potential at time t for dropping out, given survival up to time t (Kleinbaum & Klein, 2012). This means a higher hazard rate indicates a worse impact on survival. Gender was later included as a grouping variable to identify difference in hazard function by group using the log-rank test in SAS PROC LIFETEST, which identifies whether KM curves for males and females are statistically equivalent. KM plots provide the shape of each group’s hazard function, and whether and how level or shape differs across groups (Singer & Willett, 2003).

Cox regression discrete-time survival analysis was performed with SAS PROC PHREG using demographic, substance use, and leisure experience variables to predict dropout. Cox regression models are the most commonly used hazard models, and are used to describe the timing of an event, model the risk of an event’s occurrence, and the influence of predictors over time (Singer & Willett, 2003). The counting process method was used, which allowed for substance use and leisure experience predictors to be included at each interval, as time-varying covariates. Time-varying covariates provide for a more precise estimate of influence on dropout as compared to using stable predictors. For example, within the current study past month substance use at Wave 3 is connected to dropout status at the same wave. This allows for inclusion of dynamic processes, such as substance use, which normatively increases across secondary school (Randolph, Fraser & Orthner 2006).

Cox regression models were tested using three nested models (i.e., A, B, and C) sequentially including demographic and known predictors (control variables), substance use, and leisure experience predictors of dropout. Nesting models allowed for identification of the effect of the group of predictors and the additional benefit of adding in subsequent groups of predictors above and beyond the first group. Nested models were compared using the likelihood ratio test, where differences in the −2LL were compared using a χ^2^ test. A significant difference indicated that the inclusion of the group of predictors provided a better fit to the data than the previous model. Model A included only stable demographic and fixed known predictors of dropout. Model B added time-varying substance use predictors past month alcohol and tobacco use. Model C added time-varying leisure experience predictors including boredom, amotivation, intrinsic, and extrinsic motivation.

## Results

### Descriptives

Of the entire sample (*N* = 601), 48.8% (*n* = 293) met the criteria for the Dropout group and 51.2% (*n* = 308) the Complete group (see Table 1). This proportion is similar to reports from the Department of Basic Education, Republic of South Africa (2013), estimating 52% of age appropriate learners to be enrolled in Grade 12. Chi-square and *t*-test results (see Table 1) indicated significant differences related to gender, living with mother/father, previously failing a grade, number of days absent from school, past month use of both alcohol and tobacco, and level of intrinsic motivation. Being in the Dropout group was associated with being male, less likely to live with their mother or father, previously failing a grade, greater number of absences from school, higher rates of alcohol and tobacco use, and lower levels of intrinsic motivation. Preliminarily, these differences suggested a need for further investigation into the relationship between demographic, substance use, and leisure experience predictors and dropping out of school.

### Survival and Hazard Functions

**Figure 1:**
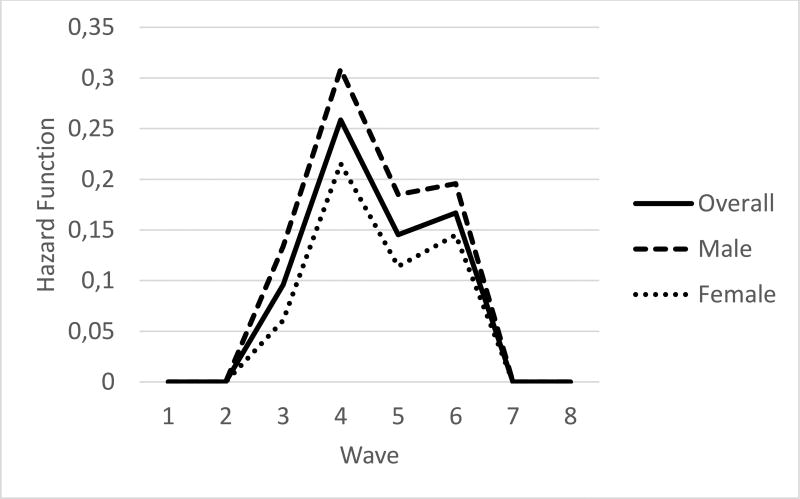
Plot of hazard function overall and by gender

We started by estimating the survival and hazard functions for dropping out. Due to the conceptualisation of dropout, survival and hazard functions were stable for Waves 1, 2, 7, and 8. At Waves 1 and 2, survival functions were 1.00 since all learners in the sample were present for these waves of data. For the entire sample, at Wave 3, the probability of survival was 0.91, Wave 4 was 0.70, Wave 5 was 0.61, and Waves 6–8 were 0.51. The hazard function for the overall sample is visually depicted in Figure 1. The hazard rate increased from Wave 3 to peak at 4 (0.10 to 0.26), decreased at Wave 5 (0.15) then slightly increased at Wave 6 (0.17).

#### Gender differences

Of those in the dropout group, 57% (*n* = 295) were male. Survival functions were compared using two homogeneity tests, which indicated significant differences in survival function by gender (log-rank Χ^2^ (1) = 15.08, *p* <.001; Wilcoxon Χ^2^ (1) = 15.67, *p* < .0001). The probability of dropping out for both males and females (given they made it to the initial interval wave) was highest at Wave 4 (end of Grade 9; 0.267 Males; 0.194 Females). By Wave 6, the proportion surviving was .437 for males and .585 for females. Figure 1 plots hazard functions by gender and visually depicts the higher hazard function of males. The hazard functions for Waves 1, 2, 7, and 8 remained constant at zero. As seen in Figure 1, the hazard rate is higher for males than for females. For both, it increased starting at Wave 2, peaked at Wave 4, decreased to Wave 5, and then demonstrated a slight increase (steeper for females) to Wave 6. The greatest hazard rate is found at Wave 4 for both males (HR = .31) and females (HR = .22). This means the hazard for males to drop out at Wave 4 is 0.31 given they have not dropped out up to that point.

### Predictors of Dropout

Nested Cox regression models were used to assess the relationship of control variables to survival time and to determine whether subsequent models adding substance use and then leisure experience would fit better than the known predictor and demographic model only. Model A results indicated gender, living with mother, and previously failing a grade significantly predicted dropout status. Parameter estimates, standard errors, and significant hazard ratios are included in Table 2 and hazard ratio (HR) and 95% confidence limits (CL) are provided within the text. As compared to males, the hazard of dropout for females was 28.2% lower (HR = 0.718; CL = 0.580–0.887). The hazard of dropout for a learner who lives with his/her mother was 29.1% lower than the hazard for a learner who does not live with his/her mother (HR = 0.709; CL = 0.524–0.961). Finally, the hazard of dropout for a learner who has previously failed a grade was 35.7% greater than a learner who has not previously failed a grade (HR = 1.357; CL = 1.097–1.678). Models B and C controlled for these demographic and known predictors.

In Model B, past month tobacco use, but not alcohol use, significantly predicted dropout status. The hazard of dropout for a learner who had used tobacco in the past month was 84.5% greater than a learner who had not used tobacco in the past month (HR = 1.845; CL = 1.486–2.290). Predictors of gender, living with mother, and previously failing a grade remained significant. Likelihood ratio test results were significant (Χ^2^ (2) = 31.69, *p* <.001) suggesting Model A be rejected.

Of the leisure predictors added in Model C, only intrinsic motivation significantly predicted dropout status. For every one unit increase in intrinsic motivation, the hazard rate decreased by 28% (HR = 0.753; CL = 0.662–0.856). Predictors significant in Model B of gender, living with mother, and past month tobacco use remained significant while previously failing a grade was no longer significant in Model C. Likelihood ratio test results were significant (Χ^2^ (4) = 18.82, *p* <.001) suggesting Model B be rejected in favour of C.

## Discussion

Survival and hazard function analyses suggest that, in this sample, differences between dropout and non-dropouts emerged. The strategy for classifying dropouts in this study, while imperfect, identified a proportion of dropout (48.8%) comparable to national statistics. Results also reflected national data, where the highest rates of dropping out for both males and females occurred at the end of Grade 9. Group comparisons between dropout and non-dropouts suggest dropouts were: more likely to be male; less likely to live with a biological parent; experienced previous academic difficulties; used alcohol and tobacco in the past month; and had lower levels of intrinsic motivation in leisure.

A comparison of the nested models predicting dropout concluded that the full model (Model C) best fit the data. In this model, predictors of school dropout included being male, not living with one’s mother, smoking cigarettes in the past month, and having lower levels of leisure-related intrinsic motivation than the non-dropout group. Of note is that failing a grade was a significant predictor of dropout until the leisure experience measures were included.

### Patterns and Predictors of School Dropout

In our sample, dropout did appear to be a process rather than an event, as suggested by Ananga (2011) and others. Constraining the data in order to focus our attention on only those who seemed to permanently dropout allowed us to more cleanly observe the temporal pattern. Although the current study does not confirm whether dropout was permanent, the literature suggests that the older youth are when they drop out, the more likely they are to stay out of school (Ananga, 2011).

The most vulnerable time for leaving school in our sample seems to be at the end of Grade Nine, which also corresponds to the end of the senior phase that allows learners to take alternative routes to further education. Due to the secondary nature of the data, we were unable to identify whether learners dropped out or left for technical or vocational pursuits. Future research ought to do so. However, learners in the current study were predominately Coloured (91%) and research suggests these youth leave school during the senior phase for complex reasons including work, substance use, and involvement with gangs (Strassburg et al., 2010).

As we continue to better understand the process of dropping out of school, we will attempt to disentangle what protective factors may be at play to prevent dropout, and what types of interventions might be effective in preventing or postponing dropping out of school. Findings from the current study suggest efforts ought to target males in particular, although clearly females had a similar vulnerability for dropping out after Grade Nine. Living with one’s mother on a consistent basis appears to be a protective factor, but this may be a very difficult target for intervention. It would be helpful to better understand why learners no longer live with their mothers (or even fathers, although that was not protective in the final model) and if the reason makes a difference in dropping out or not. For example, one might speculate that if the reason for no longer living with one’s mother was because of illness or death, this type of catastrophic reason may differentially impact someone who moved out of the house of his or her own volition, and there may be policy implications for providing care to youth in those situations. However, we are unable to determine reasons for not living with one’s mother with available data.

Preventing tobacco use is a strong concern. In this study, using tobacco is associated with school dropout, but we do not know the causality of this relation. The finding that alcohol did not significantly predict dropout status is somewhat anticipated, given the lack of consistent association found between alcohol use and dropping out. We also do not know the causal process related to experiencing intrinsic motivation to do interesting and healthy things in one’s leisure time. It is easier to make the case that those who are intrinsically motivated are more purposeful and happier in their leisure, and therefore, are less likely to drop out of school.

The role of intrinsically motivated leisure compared to extrinsically motivated leisure and being bored in leisure seems complex when interpreting our findings. The process of school leaving seems to be mostly associated with things out of the control of the learner, although one’s innate intellectual ability no doubt plays a significant role. This is consistent with the life of an adolescent, where most decisions and possible actions/behaviours are controlled by parents and societal rules. Ananga’s (2011) interviews with Ghanaian youth dropouts provide rich detail about external factors that were implicated in youth leaving school, either temporarily or permanently. These interviews also uncover how life circumstances combine to thwart academic progress and how the spiralling confluence of these external circumstances combine to lead to failing a grade and high absenteeism. Thus, what may seem to be factors in the control of the child (e.g. smoking cigarettes) may actually be artefacts of their living situation.

Given that reasoning, the fact that intrinsic leisure motivation served as a protective factor is worth considering further. The leisure context is one of the few contexts of adolescents’ life where they have the possibility for more self-determination in what they choose to do. In our previous research, we have found that those youth who report high levels of extrinsic motivation, compared to those with high levels of intrinsic motivation, have higher rates of substance use and other negative outcomes (e.g., Palen, Caldwell & Smith, 2007). In the case of school dropout, because much of dropping out seems caused by the interplay of external reasons, it is possible that extrinsic forms of leisure motivation were not important, because these youth already felt high levels of being externally controlled. By way of contrast, those who for some reason felt intrinsically motivated were more likely to persist in school. Perhaps these youth possessed characteristics or skills that allowed them to navigate their world in a more self-determined manner in general. For example, learners who experience intrinsic motivation in their leisure time may also possess characteristics (e.g. higher levels of cognitive engagement as noted by Fredricks, Blumenfeld & Paris, 2004) that facilitate similar experiences within the school context. These are all speculations in need of empirical examination.

### Life Orientation Curriculum

The Life Orientation Curriculum aims to develop skills, knowledge and values for personal, social, intellectual, emotional and physical growth of learners and the focus is on self-motivation and making informed choices and decisions in life (Department of Basic Education, Republic of South Africa, 2011a). As such, this learning area has the potential to serve as a protective factor by helping learners gain skills to reduce substance use and engage in healthy leisure behaviours. One of the goals of Life Orientation is to promote movement and physical development; however, this could be expanded to include exploration and participation in leisure and recreation activities and serve as a programme which re-engages learners to prevent dropout in secondary school.

Educating learners in ways to become involved in personally meaningful and interesting leisure activities could be coupled with teaching learners to be in touch with why they do certain things during the day. Helping learners understand about personal motivation more deeply and in a more nuanced way (e.g. learning about intrinsic versus introjected motivation) might help them develop personal control in some aspect of their life, thereby mitigating feelings of being overwhelmingly externally controlled.

## Conclusions and Future Directions

Results from the current study provide a new perspective (e.g. one that accounts for leisure) on the issue of SA adolescent dropout. Rather than a distinct event, dropout has been depicted as a process of disengagement over time from the educational system. This is evidenced in Freudenberg and Ruglis’ identification of 39 individual, family, community, and school factors associated with dropout, leading the authors to conclude “the multiple factors associated with dropout rates suggest that no single type of intervention can end our nation’s dropout crisis” (2007:2). Although Freudenberg was focusing on the US, the same conclusion could be made about dropout globally.

A number of limitations exist within the current study. First, we are using participation at each survey administration as a proxy for dropout and have no confirmation as to whether learners actually dropped out of school. One attempt to address this issue was by conceptualising dropout as learners who participated at Waves 1 and 2 and were absent for Waves 7 and 8 and choosing the comparison group as those that attended each survey administration. Additionally, because the original study did not directly target dropout, we were unable to incorporate additional factors known to be associated with dropout (e.g., school climate, peer academic aspirations) into analyses. For example, although we did have a measure of race, the sample was predominantly Coloured and therefore precluded further racial comparisons. Despite these limitations, the current study provides valuable information on what contributes to learners leaving school prematurely.

There were a number of interesting findings from the current study. Given the group comparison results, we were surprised that number of days absent from school and alcohol use (although somewhat less surprised) did not predict dropping out. Furthermore, we were surprised that the addition of leisure experience items in Model C resulted in failing a grade to drop out of the model, given that this variable is known to be strongly associated with dropout. Finally, we expected more leisure experience variables to predict dropout status due to our prior work on leisure within this geographical context. We recommend future research further addresses the association between educational disengagement, substance use, and leisure experience. Qualitative data would be especially useful in understanding the lived experience of dropout, although we acknowledge that tracking down dropouts may be challenging. Finally, from a prevention perspective, results suggest it is warranted to develop comprehensive prevention programmes or enhancing the current Life Orientation curriculum in order to target risk behaviour and leisure, given that high intrinsic motivation served as a protective factor against dropout status.

## Figures and Tables

**Table 1:** Baseline descriptives for dropout predictors of dropout Included in analyses for dropout and non-dropout groups

Predictor	Dropout Group (*n* = 293)	Non-Dropout Group (*n* = 308)	Χ^2^ Difference Test or *t*-Test Results, *p* value
Gender			
Female	43.3% (*n* = 127)	58.1% (*n* = 179)	13.11, *p* < .001
Living with parent			
Mother	87.7% (*n* = 257)	94.1% (*n* = 290)	7.62, *p* < .01
Father	63.1% (*n* = 185)	72.7% (*n* = 224)	6.35, *p* < .05
Previously Failed a Grade	52.9% (*n* = 155)	37.0% (*n* = 114)	15.33, *p* < .0001
Number of Days Absent from School *M* (*SD*)	8.61 (10.38)	6.59 (7.64)	*t* = −2.70, *p* < .01
Substance Use (Past Month)			
Alcohol	15.1% (*n* = 44)	7.4% (*n* = 23)	8.82, *p* < .01
Tobacco	27.7% (*n* = 81)	48.4% (*n* = 31)	30.84, *p* < .0001
Free Time Experience *M* (*SD*)			
Boredom	1.52 (0.98)	1.51 (0.92)	*t* = −0.20, *p* =.844
Amotivation	1.62 (1.02)	1.57 (0.99)	*t* = −0.63, *p* =.531
Intrinsic Motivation	2.77 (0.82)	3.01 (0.66)	*t* = 3.84, *p* < .0001
Extrinsic Motivation	1.91 (0.99)	1.97 (0.92)	*t* = 0.76, *p* =.448

*Note*. *N* = 601. *M* = mean; *SD* = standard deviation.

**Table 2:** Results of fitting Cox regression nested models to dropout data

	Model A	Model B	Model C
Parameter Estimates (Standard Errors)			
Significant Hazard Ratios			
Gender	−0.332** (0.108)	−0.367*** (0.108)	−0.359** (0.109)
	0.718	0.693	0.698
Mother	−0.343* (0.155)	−0.315* (0.149)	−0.306* (0.149)
	0.709	0.729	0.737
Father	−0.184 (0.113)	−0.174 (0.112)	−0.159 (0.112)
Fail	0.304** (0.109)	0.247* (0.109)	0.184 (0.112)
	1.357	1.280	
Absent	−0.008 (0.004)	0.003 (0.005)	0.005 (0.005)
	1.008		
Past Month Alcohol Use		0.141 (0.115)	0.183 (0.115)
Past Month Tobacco Use		0.612*** (0.110)	−0.605*** (0.109)
		1.845	1.831
Boredom			−0.004 (0.068)
Amotivation			−0.060 (0.068)
Intrinsic Motivation			−0.284*** (0.065)
			0.753
Extrinsic Motivation			0.009 (0.068)

Goodness-of-Fit			
−2LL	3599.011	3567.315	3548.499
LR statistic	29.830	61.344	76.826
*n* parameters	5	7	11
*p*	< .0001	< .0001	< .0001
AIC	3609.011	3581.315	3570.499
BIC	3627.412	3607.076	3610.980

Likelihood Ratio Tests		Model A vs. B	Model B vs. C
−2LL χ^2^		31.696*** (*df* = 2)	18.816*** (*df* = 4)

*Note*.

* *p* < .05;

** *p* < .01;

*** *p* < .001.

Breslow method for ties.
